# HIV RNA Suppression and Immune Restoration: Can We Do Better?

**DOI:** 10.1155/2012/515962

**Published:** 2012-03-25

**Authors:** Marilia Rita Pinzone, Michelino Di Rosa, Bruno Cacopardo, Giuseppe Nunnari

**Affiliations:** ^1^Division of Infectious Diseases, Department of Clinical and Molecular Biomedicine, University of Catania, Catania 95125, Italy; ^2^Department of Microbiology and Immunology, Jefferson Medical College, Thomas Jefferson University, Philadelphia, PA 19107, USA

## Abstract

HAART has significantly changed the natural history of HIV infection: patients receiving antiretrovirals are usually able to control viremia, even though not all virological responders adequately recover their CD4+ count. The reasons for poor immune restoration are only partially known and they include genetic, demographic and immunologic factors. A crucial element affecting immune recovery is immune activation, related to residual viremia; indeed, a suboptimal virological control (i.e., low levels of plasma HIV RNA) has been related with higher levels of chronic inflammation and all-cause mortality. The sources of residual viremia are not yet completely known, even though the most important one is represented by latently infected cells. Several methods, including 2-LTR HIV DNA and unspliced HIV RNA measurement, have been developed to estimate residual viremia and predict the outcome of antiretroviral therapy. Considering that poor immunologic responders are exposed to a higher risk of both AIDS-related and non-AIDS-related diseases, there is a need of new therapeutic strategies, including immunomodulators and drugs targeting the latent viral reservoirs, in order to face residual viremia but also to “drive” the host immunologic responses.

## 1. Introduction

The introduction of highly active antiretroviral therapy (HAART) has determined a significant reduction in morbidity and mortality of people living with human immunodeficiency virus (HIV) [1]. The majority of individuals taking HAART experience HIV RNA suppression below the detection limit of clinical assays (usually 20–50 copies HIV RNA/mL plasma) [2, 3]. However, despite suppressing viremia, HAART cannot eradicate HIV: residual low level viremia (LLV) can indeed be detected in most patients with ultrasensitive assays, because of the persistency of viral reservoirs and “sanctuary sites” not fully affected by HAART [4–7]. In addition to plasma HIV RNA, several protocols have been developed to estimate the burden of viral replication, including 2 “long terminal repeat” (LTR) HIV DNAs, a marker of recent cellular infection, and multispliced (MS) and unspliced (US) HIV RNA quantification [8]. Of note, not all virologically suppressed patients are able to recover their CD4+ T-cell count, thus representing a great concern because of the risk for opportunistic infections.

The reasons for defective immune restoration are not fully understood: reduced CD4+ T-cell recovery has been associated with older age [9–14], higher HIV RNA before HAART [10, 12, 15–19], lower baseline CD4+ count [10–12, 20–22], bone marrow [23, 24], and thymic dysfunction [25–27]; genetic factors, including CCR5 polymorphism [28], some antiretroviral drugs [29–31], and immune activation [32] has also been related with impaired immune restoration.

In this paper, we first describe the mechanisms which may affect immune restoration, focusing on the role of immune activation and residual viremia. We briefly outline the main sources of LLV and the most commonly used assays to identify latently infected cells and we report the most recent evidence about the clinical implications related to LLV. We then summarize the potentialities of new therapeutic options, including immune therapy and “reactivation strategies,” in reconstituting immune functions of HIV-infected subjects.

## 2. Definition and Timing of Immune Restoration

The increase in peripheral CD4+ T cells observed during HAART occurs in three distinct phases: during the first 3–6 months of HAART, the significant increase (20–30 cells/*μ*L monthly) in circulating naive and memory CD4+ T cells may be explained by the redistribution of T cells from the lymphoid tissues to the blood [33–35]; viral load suppression, with the subsequent decrease in immune activation, has been shown to downregulate the expression of adhesion molecules on the surface of T cells, such as intercellular adhesion molecule-1 (ICAM-1) and vascular cell adhesion molecule-1 (VCAM-1), which are responsible for T-cell trapping in the lymphoid organs. This mechanism results in T-cell dismissal in the blood [36]. The second phase (5–10 cells/*μ*L monthly, until the end of the second year of HAART) and the third phase (2–5 cells/*μ*L monthly, until at least the seventh year of antiretroviral therapy) of immune restoration are due to several mechanisms, globally leading to the rise in T cells, especially naive ones [10, 37, 38]: the stimulation of thymic lymphopoiesis and the proliferation of residual CD4+ T cells are the main causes of immune restoration [39]; lifespan increase of CD4+ T cells may also account for T-cell recovery under HAART, especially in older subjects, who physiologically have a reduced thymic function [40]. Of note, these potentially compensatory responses to HIV infection do not have the same immunological meaning, considering that *de novo* proliferation of CD4+ T cells may potentially restore a complete T-cell repertoire; by contrast, the increased proliferation and survival of residual CD4+ T cells, even if able to apparently guarantee T-cell recovery, is not associated with a good quality of immune reconstitution, because of its inability to reconstitute a complete T-cell repertoire.

In most studies, a strong correlation between the magnitude of the change in plasma HIV RNA and the increase in circulating CD4+ T cells has been described. Le Moing et al. [37] reported a significant association between the long-term slope of CD4+ T cells and the variation of plasma HIV RNA levels when studying a large cohort of HIV-infected patients at the initiation of a protease-inhibitor- (PI-) containing antiretroviral regimen. In fact, the long-term slope was 2.5 cells/mm^3^/month higher in patients who had plasma HIV RNA levels of less than 500 copies/mL at month 4 (*P* < 0.001), in comparison with those having no virological response. Nevertheless, this increase in CD4+ T-cell count was significantly attenuated after occurrence of a rebound in plasma HIV RNA > 500 copies/mL, thus suggesting the importance to achieve and maintain a good virological control. Pretreatment HIV RNA levels have been significantly correlated with CD4+ T-cell recovery in several studies [14, 37–39]: as previously noticed, this observation may be due to the fact that higher plasma HIV RNA levels associate with greater numbers of CD4+ T cells being sequestered within lymphatic tissues, resulting in a greater redistribution of cells after HAART-induced viral suppression.

It is interesting to note that CD4+ T-cell recovery under HAART does not lead to the full restoration of humoral and cellular immune functions. Indeed, a number of studies have described functional impairment of both innate and acquired immune responses despite effective HAART. Chehimi et al., for instance, observed only a partial recovery of functional plasmacytoid dendritic cells (PDCs) and natural killer (NK) cells after 52 weeks of suppressive HAART [41]. In this study, patients were divided into two groups, one composed of subjects achieving rapid viral load suppression (HIV RNA < 50 copies/mL by week 12), the other composed of subjects with delayed viral suppression. Subjects with delayed viral suppression had a higher baseline viremia (29.254 copies/mL *versus* 4.134 copies/mL); moreover, baseline viral load was a negative predictor of PDCs recovery after 52 week of HAART (*r* = −0.47,   *P* = 0.08). Restoration of total NK cells was incomplete even after 52 weeks on HAART when considering the whole HIV-positive cohort (73 cells/*μ*L *versus* 122 cells/*μ*L in controls). Furthermore, cytokine-induced IFN-gamma production by NK cells has been shown to be similarly impaired in asymptomatic, viremic, and HAART-suppressed HIV-positive subjects [42]. Stone et al. [43] found that CD4+ T cells from HIV-infected patients had increased expression of the coinhibitory protein cytotoxic T-lymphocyte antigen-4 (CTLA-4) and decreased expression of the costimulatory protein CD28, even in presence of increased CD4+ T-cell count and great control of HIV viral load by HAART. CTLA-4 and CD28 play a crucial role in T-cell activation after engagement by CD80/CD86 on antigen-presenting cells, so that their dysregulated expression on T cells may contribute to impaired T-cell functions.

There is not a consensus definition of immunologic nonresponder individuals: some authors described patients on HAART whose CD4+ T-cell count remained below a critical threshold, ranging between 350 and 500 cells/*μ*L, over an extended period of time (more than 4–7 years in most studies), as poor immune responders, in which viro-immunologic dissociation implies a greater risk of AIDS-related and non-AIDS-related illnesses [44–46]; some others analyzed immune recovery over a shorter period of time, usually 6–12 months of HAART, defining as immunologic nonresponders those patients whose CD4+ T-cell count increase was <30% and total CD4+ T-cell count ≤ 200 cells/*μ*L [47].

## 3. Risk Factors for Impaired CD4+ T-Cell Recovery

**Figure 1:**
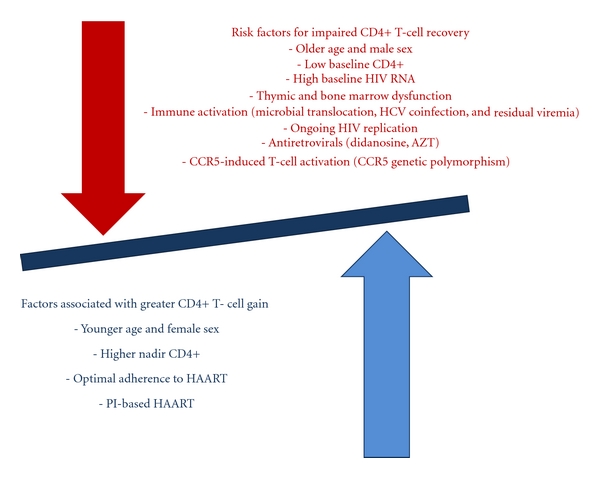
Factors affecting immune restoration in patients on HAART.

Immunologic nonresponse despite effective HAART appears to have a multifactorial origin, since a number of host-related and HIV-related factors contribute to a suboptimal immune reconstitution [48] (Figure 1). Some demographic characteristics, like male sex and older age, have been associated with reduced CD4+ T-cell gain: these findings may be explained by the fact that thymic output is higher in women and younger subjects and it is closely associated with *de novo* production of CD4+ T cells [49]. In fact, impaired central regeneration of T cells, consistent with altered thymopoiesis, has been reported as a key determinant of poor immune reconstitution. T-cell receptor (TCR) excision circles (TRECs) have been used as a surrogate marker of thymic output, because they represent sequences of extrachromosomal DNA which are not replicated during T-cell divisions and therefore are not present in the progeny. In subjects with reduced immune restoration, a lower level of TRECs in T cells has been found by some authors [50, 51]; in addition, a lower percentage of recent thymic immigrant CD4+ T cells (CD31+%), has been described in immunologic nonresponders in comparison with immunologic responders [52], thus supporting, again, the contribute of thymic exhaustion to poor CD4+ gain despite suppressive HAART [25–27]. It remains to be established if it is the thymus itself to be unable to respond to thymopoietic signals, for example, IL-7, or if it is the insufficient production of thymopoietic molecules to compromise thymic-dependent immune recovery. IL-7 is a potent pleiotropic cytokine implicated in both thymopoiesis and peripheral homeostasis. IL-7 is constitutively produced by stromal cells from the bone marrow and thymus. In the thymus, it supports the viability and the expansion of early thymocytes; at the peripheral level, it costimulates T cells, thereby initiating their proliferation and survival. Furthermore, it has been shown to increase CD8+ T-cell cytotoxicity and to promote NK-cell functions [53]. IL-7 production is increased in lymphopenic conditions [54]. In fact, when studying a cohort of 168 HIV-positive patients, Napolitano et al. found higher IL-7 levels to be independently associated with lower CD4+ T-cell count (*P* = 0.0001) and higher plasma HIV RNA levels (*P* = 0.002). Consistent with the findings of the cross-sectional study, the longitudinal analysis of a smaller cohort of 11 HIV-infected patients, observed over 6–25 months, confirmed the increase in IL-7 levels to be significantly associated with a decrease in the CD4+ T-cell count (*ρ* = −0.64; *P* = 0.03) but not with changes in viral load anymore (*ρ* = −0.41; *P* = 0.21). Despite similar IL-7 plasma levels in immunologic responders and nonresponders, it has been shown that patients with poor immune recovery are characterized by reduced IL-7 receptor expression on different T-cell populations, thus potentially limiting the compensatory effect of higher IL-7 levels [55]. Female sex hormones have been shown to exert an antiapoptotic role on neutrophils [56]; it may be speculated a similar function on CD4+ T cells, which may at least partially explain the greater immune restoration found in women in comparison with men. A more direct link between T-cell recovery and gender has been recently described by Olsen and Kovacs, who demonstrated that the thymus is a target for androgen hormones, as suggested by the observation of increased thymic T-cell output in hypogonadal men [57]. The role of genetics has been emphasized by several authors: the stimulation of C-C chemokine receptor type 5 (CCR5) on CD4+ T-cell surface has been associated with T-cell activation [58]; in subjects carrying a CCR5 polymorphism, determining CD4+ T-cell activation, the polymorphism has been found to be predictive of immunologic response [28]. Multiple studies reported that patients receiving PI-based regimens had a better T-cell recovery [59–62], possibly because PIs may restore T-cell proliferative responses [63] and activate antiapoptotic pathways [64]. The administration of other antiretroviral drugs has been reported to have a deleterious effect on CD4+ T-cell gain: AZT, for instance, has been shown to be toxic for hematopoietic progenitors [29], didanosine to block T-cell proliferation and differentiation [30].

Immune activation is considered a key element in determining poor immune restoration [23, 32, 55, 65–67]: the immune system of HIV-infected subjects has to cope with a massive T-cell apoptosis [68, 69], which appears closely associated with the continuous stimulatory effect of proinflammatory cytokines and viral antigens [70]. The depletion of CD4+ T cells in mucosal lymphoid tissues is considered a key element of immune activation, because it leads to the disruption of the mucosal barrier in the gut, whose physiological function is to prevent microbial translocation from the gut to the systemic immune system [70–73]. Microbial translocation results in elevated plasma levels of bacterial 16s DNA and lipopolysaccharide (LPS) [73, 74], which have been directly correlated with reduced CD4+ T-cell recovery after starting HAART [66, 73, 74]. Coinfection with HCV [75–77] or Herpesviridae (EBV, CMV) reactivation [78, 79] have also been associated with immune activation and poor CD4+ T-cell recovery.

## 4. Residual Viremia, HIV Latency, and Poor Immune Restoration

Residual viremia has been shown to trigger immune activation [48]: it is not yet well known if residual LLV represents the result of ongoing cycles of viral replication or if it is caused by the release of virus from stable HIV reservoirs [80]. As refers to the first hypothesis, some authors have recently shown that there is not evolution in HIV genome during HAART, thus implying the absence of ongoing cycles of productive viral replication. Furthermore, the characterization of rebounding virus during structured therapeutic interruptions has shown no evidence of evolution when compared with pretreatment samples [81]; analogously, some studies reported that HAART intensification with the addition of raltegravir, in patients on suppressive regimens, did not significantly decrease viremia, thus suggesting, again, the absence of ongoing cycles of replicating virus [82–85]. Further support to this observation emerged from earlier studies, showing that the level of persistent viremia was not related to treatment regimen but to pretreatment viral load; all effective HAART regimens suppressed HIV viral load to the same average level, a level that was not determined by the regimen itself, but by the size of the viral reservoirs [86]. On the other hand, some authors have reported opposing results: the presence of extrachromosomic HIV DNA, including circular DNA containing “long terminal repeat” sequences (LTR), a putative marker of recent cell infection, has been found in virologically suppressed patients [87]; likewise, an increase in the amount of 2-LTR forms of HIV DNA in the peripheral blood mononuclear cells (PBMCs) of patients under treatment intensification with raltegravir supported the idea of the persistency of ongoing replication [88].

The presence of long-lived latently infected cells is generally considered the main cause of LLV. HIV latency occurs in resting central memory (T_cm_) and transitional memory (T_tm_) CD4+ cells [89], but also in astrocytes [90, 91], monocyte-macrophages [92], naive T cells [93, 94], and thymocytes [95]. Latently infected cells can be detected in blood and tissues, such as the gastrointestinal tract [96], the central nervous system [91, 97], the genital tract [98–100], which represent “sanctuary sites”, where HAART poorly penetrates. When activated, latently infected CD4+ T cells can release the virus in the blood, even if antiretrovirals are able to prevent new rounds of infection; during HAART, these cells decay very slowly, with an average half-life of 44 months, so that under current treatment it will take over 60 years to deplete this reservoir [101]. The pretreatment HIV-1 DNA level in PBMCs has been identified as a relevant marker in predicting residual viremia for patients receiving an identical HAART regimen [4], as cell-associated HIV DNA is considered a good surrogate marker of the total number of latently infected cells [102]. Quantification of cell-associated unspliced (US) and multiply spliced (MS) HIV RNA has been proposed as an helpful tool to estimate residual productively infected cells and to predict the virological outcome of therapy in patients on HAART: Pasternak et al. demonstrated that the level of HIV US RNA in PBMCs, as measured in HAART-treated patients with undetectable viremia, strongly correlated with the likelihood to experience HAART failure. In fact, median US RNA levels were significantly higher in patients who underwent HAART failure (0.43 log_10_ difference, *P* = 0.0015) in comparison with those who remained virologically suppressed and inversely correlated with baseline CD4+ T-cell count; furthermore, in multivariate analysis, after adjusting for baseline CD4+ T-cell count, prior HAART experience and particular HAART regimens, the maximal US RNA level under therapy was the best independent predictor of subsequent therapy failure (adjusted odds ratio [95% CI], 24.4 [1.5–389.5], *P* = 0.024) [103].

Several studies have shown that the persistency of residual viremia represents a continuous proinflammatory stimulus for the immune system, causing immune activation and chronic inflammation. Of interest, a recent work of d'Ettorre et al. [104] has found out the association of HIV persistence in the gut mucosa of HAART-treated subjects with immune activation and microbial translocation: in fact, the level of HIV DNA in the gut correlated with plasma level of LPS and with the levels of expression of the activation marker CD38 on CD8+ T cells. In the SMART trial, levels of C-reactive protein (CRP), Interleukin (IL)-6, and D-Dimer remained elevated in HIV-infected subjects, despite suppressive HAART [105]; furthermore, IL-6 and D-Dimer were strongly associated with mortality, even in patients achieving viral suppression [106]. Contrasting results were recently published by Eastburn et al. [107]. The authors explored the association of LLV with inflammation, increased coagulation, and all-cause mortality and they unexpectedly found little association of LLV with CRP, IL-6 and fibrinogen: in fact, CRP levels did not correlate with HIV viral load, while increasing HIV RNA was associated with higher IL-6 and fibrinogen levels, but only for subjects whose viral load was above 10.000 copies/mL. As refers to IL-6, this association was attenuated by ~50% after adjusting for CD4+ T-cell count. In addition, HIV RNA was not associated with mortality risk over 5 years, after adjustment for CD4+ T-cell count, cardiovascular risk factors, and inflammation. These contrasting data suggest the need for more studies, properly designed to identify the most reliable coagulopathic/inflammatory markers associated with HIV ongoing replication/persistence and to further explore whether LLV predisposes to increased inflammation and all-cause mortality. Furthermore, in addition to cross-sectional plasma HIV RNA measures, some authors have investigated the use of other biomarkers, which may be able to better describe the patient longitudinal exposure to HIV burden and to potentially work as a proxy measure of cumulative inflammation and immune system activation [108, 109]. Mugavero et al. have recently proposed viremia copy-years, a time-varying measure of cumulative plasma HIV exposure, as an independent predictor of mortality, even after adjusting for most recent CD4+ T-cell count, thus substantiating the role of HIV replication in accelerating disease progression, independently of its effects on peripheral CD4+ T-cell depletion [108].

## 5. Potential Strategies to Reduce Residual Viremia and Enhance Immune Restoration

**Figure 2:**
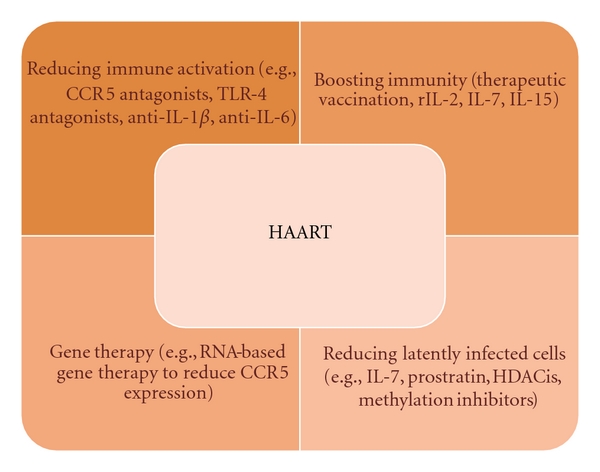
Therapeutic options for increasing CD4+ T-cell recovery. HAART obviously remains the milestone of any treatment; in addition, other potential strategies, targeting some aspects associated with poor immune restoration, are here reported.

Considering that functional and numerical normalization of CD4+ T-cells appears to be fundamental to prevent both AIDS-related and non-AIDS-related morbidity and mortality, there is a need of new therapeutic strategies for immunologic nonresponder patients (Figure 2).

We have previously highlighted the crucial role of immune activation in causing poor CD4+ T-cell gain in patients receiving HAART; therefore, strategies aiming at reducing immune activation would be worthy to enhance immune restoration. An attempt to suppress polyclonal T-cell activation has been made by some authors using immunosuppressive drugs, such as hydroxyurea, corticosteroids, cyclosporine A, mycophenolate, with no effective results, even considering that immunosuppressive adverse effects may overwhelm the theoretical benefits of reduced T-cell activation [110]. More encouraging results have been associated with the use of maraviroc, a CCR5 antagonist, which has been shown to reduce the levels of activated CD4+ and CD8+ T cells [111, 112] and to induce a greater increase in CD4+ T-cell count in treatment naive patients at week 48 and 96, in comparison with efavirenz [113]. The anti-inflammatory and immunomodulatory functions of statins have recently been evaluated by De Wit et al., who have shown atorvastatin to significantly reduce the level of immune activation, measured by CD8+/CD38+ percentage, but not to affect highly sensitive CRP levels or CD4+ T-cell response in HAART-suppressed patients [114]. As refers to microbial translocation, the use of probiotics has been suggested to favorably modify the balance between “good” and “bad” intestinal flora; furthermore, the use of inhibitors of bacterial product-mediated effects (antagonists of toll like receptor-4, the receptor for LPS [115]) or inhibitors of proinflammatory cytokines (anti-IL-1*β*, anti-IL-6 or antitumor necrosis factor (TNF)-*α* [116]) is another option currently under evaluation. The antiviral and anti-inflammatory properties of the antimalarial drug hydroxychloroquine (HCQ) have recently been analyzed in a cohort of 20 HAART-treated immunologic nonresponders by Piconi et al. [117], who studied the impact of HCQ (400 mg/day for 6 months) on immune activation: HCQ significantly reduced plasma LPS, LPS/TLR-mediated signal transduction and IL-6/TNF-*α* production; furthermore, it increased percentages of circulating CD4+ T cells. These effects were mostly retained two months after therapy interruption. However, a trend, but not a significant increase in CD4+ T-cell count was observed, thus indicating that more studies are needed to check the immunomodulating potentialities of HCQ. Some clinical trials are currently in progress (ACTG A5258, http://clinicaltrials.gov/ct2/show/NCT00819390; CTN246, http://www.hivnet.ubc.ca/clinical-studies/canadian-hiv-trials-database/ctn-246/).

Strategies to restore the regenerative capacity of immune system include the administration of recombinant interleukin-2 (rIL-2) and IL-7. Endogenous IL-2 is produced by activated CD4+ T cells and it is important for antigen processing and induction of CD8+ T-cell cytotoxic activity; in HIV-positive subjects, IL-2 levels are lower, so that CD8+ cytotoxicity is impaired and the virus may easily escape from the host immune system. Some clinical trials demonstrated the efficacy of one year-treatment with rIL-2 plus HAART *versus* HAART alone in determining CD4+ T-cell gain [118, 119]; furthermore, the benefits of rIL-2 administration in terms of CD4+ count were maintained for over 95 months in another follow up study [120]. First, rIL-2 administration may help facing CD4+ T-cell loss, mainly through the expansion of the naive CD4+ compartment; it does not seem to be a mere matter of quantity but also of quality: in fact, there was not only a numerical increase but also a significant rise in functionally competent CD4+ CD28+ T cells, being CD28 a costimulatory molecule, whose expression is needed for antigenic presentation [121, 122]. In addition, rIL-2 may promote the proliferation of CD8+ T cells [123] and inhibit HIV replication in macrophages [124]. Despite the significant increase in CD4+ T-cell count, the ESPRIT and SILCAAT study, two recent prospective, randomized, controlled, phase III studies exploring the clinical benefits of intermittent subcutaneous rIL-2 with HAART *versus* HAART alone, have shown no significant differences in HIV RNA levels and no clinical benefits to the patients in the rIL-2 arm [125, 126]. As refers to IL-7, a recent trial has demonstrated a sustained increase in T cells after administration of recombinant human (rh) IL-7 to HIV-infected patients [127], because of both increase in thymopoiesis as well as a direct increase in the magnitude of antigen-driven peripheral T-cell expansion [128].

Another potential immunomodulatory strategy is associated with NK-cell capability to exert a direct cytotoxic effect and to produce soluble factors able to control HIV replication [129]. From this perspective, some authors have evaluated the immunological effects of IL-15, a pleiotropic cytokine which is involved in NK-cell proliferation and survival [130]. D'Ettorre et al. have recently reported that the *in vitro* challenging of NK cells with IL-15 was able to significantly reduce the level of viral p24 antigen in cocultured infected CD4+ T cells and PBMCs media. Furthermore, IL-15 stimulated NK cells reduced HIV DNA viral load both in purified CD4+ T cells and PBMCs [131]. In a previous study of the same group, IL-15 priming was shown to induce a significant increase of IFN-gamma production in both viremic and aviremic HIV-positive patients [132]. Tarkowski et al. [133] found that the expression of IL-15, but not of its receptor IL-15R*α*, was significantly higher in the CD14+ monocytes of long-term nonprogressors than in those of HIV-1 progressors or healthy controls; differences in IL-15 expression between patients with different courses of HIV infection may be indicative of a potential use of this cytokine as a therapeutic agent.

Many strategies have been explored to eradicate residual viremia in subjects on HAART: latently infected cells do not differ from uninfected cells, apart from the presence of integrated HIV DNA but, when activated, these cells may become vulnerable to immune-mediated killing and to antiretrovirals, so that a current “hot topic” is focused on reactivating latently infected cells, in order to induce integrated HIV expression; for example, IL-7 has been shown to induce productive infection from latently infected CD4+ T cells *in vitro*, through the activation of the Jak-Stat pathway [127, 134, 135] and it is currently ongoing clinical trials, to determine if it may be effective to reduce *in vivo* latent reservoirs (ERAMUNE, http://www.clinicaltrials.gov). It has been recently shown that the stimulation of CD4+ T cells from viremic donors with IL-7 or IL-15 was able to induce in both cases viral production in productively infected cells, even if these cytokines showed a different impact on the maintenance of latently infected CD4+ T cells: in fact, IL-7 had a major effect on homeostatic proliferation of cells harboring integrated HIV DNA, whereas IL-15 promoted the differentiation of T_cm_ CD4+ T cells and increased the percentage of short-lived effector memory (T_em_) T cells [136].

The upregulation of cellular transcription has been suggested as another strategy to induce HIV genome expression and reverse latency, for instance by promoting histone acetylation [137]. Histone deacetylase inhibitors (HDACis), such as valproic acid, sodium butyrate, and suberoylanilide hydroxamic acid (SAHA), have been shown to enhance cellular transcription, including HIV LTR [138]; however, some retrospective studies failed to demonstrate a significant reduction of latent reservoirs when administering valproic acid [139–141]. Due to the fact that active nuclear factor-kappa B (NF-*κ*B) is a positive regulator of HIV expression, the use of the NF-*κ*B activator prostratin, in combination with HDACis, has been shown to enhance HIV transcription *in vitro *[142, 143]. Mehla et al. have recently reported the *in vitro* capability of bryostatin, a Protein Kinase C (PKC) agonist, to reactivate latent viral infection in monocytic and lymphocytic cells, mainly because of PKC-induced activation of NF-*κ*B [144]. In addition, considering that DNA methylation prevents HIV expression, the use of methylation inhibitors, such as decitabine, has been proposed to eliminate HIV from latently infected resting CD4+ T cells [145, 146]. Anyhow, the *in vitro *efficacy of these families of compounds needs to be tested first in animal models and then in well-designed and well-tolerated clinical trials, to understand if the “reactivation strategy” may also work *in vivo*.

Recently, it has been suggested that compounds able to promote the differentiation of the long-lived T_cm_ and T_tm_ CD4+ T cells, which play a main role in HIV persistence [89], into T_em_-like phenotypes, may contribute to the restriction of the viral reservoir. The gold-based compound auranofin, which acts through the induction of reactive oxygen species, has been shown to accelerate CD4+ T-cell turnover, thus inducing long-lived memory cells to progress to short-lived phenotypes and die [147].

Finally, therapeutic vaccination has been evaluated over the last years, with mixed results, as a strategy aiming at boosting immunity to HIV, generating HIV-specific CD4+ and CD8+ T-cell responses, possibly able to control viral replication and enhance immune recovery [148, 149].

## 6. Conclusions

HAART has profoundly changed the natural history of HIV infection, but a good suppression of HIV does not mean viral elimination. We are still looking for therapeutic approaches able to eradicate the virus; the persistency of latently infected cells and latent reservoirs represents indeed a major challenge for the virologists and clinicians, considering that residual LLV works as a trigger for immune activation and increased T-cell apoptosis.

Furthermore, not all virologically suppressed patients are able to effectively recover their CD4+ T-cell count, thus exposing immunologic nonresponders to a significant risk of both AIDS-related and non-AIDS-related morbidities. Despite the ever-growing number of new available antiretrovirals, giving us new “weapons” to control viremia, the approach to patients with poor immune recovery is challenging; immune modulation, together with drugs targeting the latent reservoirs, appear the most encouraging options. Further studies are needed to better understand the mechanisms that influence immune restoration and to validate *in vivo *the evidence coming from* in vitro *studies.
